# Dysfunctional Attitudes Toward Motherhood and Depressive Symptoms in Portuguese Pregnant Women During COVID-19 Pandemic: the Mediating Roles of Self-Compassion and Mindful Self-Care

**DOI:** 10.1007/s12671-022-02049-5

**Published:** 2022-12-29

**Authors:** Sandra Xavier, Mariana Branquinho, Raquel Pires, Helena Moreira, Maria Coelho, Anabela Araújo-Pedrosa

**Affiliations:** 1 Center for Research in Neuropsychology and Cognitive and Behavioral Intervention, Faculty of Psychology and Education Sciences, University of Coimbra, Coimbra, Portugal; 2 Clinical Psychology Service & Department of Gynecology, Obstetrics, Reproduction and Neonatology, Centro Hospitalar e Universitário de Coimbra, Coimbra, Portugal

**Keywords:** COVID-19, Dysfunctional attitudes toward motherhood, Depressive symptoms, Self-compassion, Mindful self-care, Pregnancy

## Abstract

**Objectives:**

There is evidence that the coronavirus disease 2019 (COVID-19) pandemic had a negative impact on the mental health of pregnant women, such as an increase in the prevalence of depression. Given the negative consequences that depressive symptoms can pose for pregnant women, it is crucial to understand how cognitive processes influence women’s depressive symptoms during the COVID-19 pandemic among this specific vulnerable population. The present study aimed to examine the relationship between pregnant women’s dysfunctional attitudes toward motherhood and their depressive symptoms, considering the mediating roles of self-compassion and mindful self-care.

**Method:**

A cross-sectional online survey was conducted in Portugal between October 2020 and April 2021. A sample of 244 pregnant women answered a set of questionnaires to assess dysfunctional attitudes toward motherhood, depressive symptoms, self-compassion, and mindful self-care.

**Results:**

More dysfunctional attitudes toward motherhood were associated with higher levels of depressive symptoms, and this relation was mediated through self-compassion and mindful self-care.

**Conclusions:**

These results highlight that self-compassion and mindful self-care are important psychological mechanisms and the importance of considering them in psychological interventions to prevent and/or treat depressive symptoms in pregnant women with dysfunctional attitudes toward motherhood during stressful events such as a pandemic.

The COVID-19 pandemic quickly expanded worldwide, becoming a public health emergency. Several studies have pointed out that the pandemic has affected the mental health of several populations (Rajkumar, 2020; Wang et al., 2020), particularly vulnerable ones such as women in the perinatal period (Liu et al., 2021; Motrico et al., 2020). The COVID-19 pandemic led to dynamic changes over time in family and community life, as well as in the health care services provided to women in the perinatal period. The pandemic’s effects on women’s mental health occurred across different phases of the pandemic and there will probably be lasting effects after its end (Brooks et al., 2020; Caparros-Gonzalez et al., 2020). However, little is known about the psychological mechanisms that may explain women’s adjustment to pregnancy under extreme public health crises such as the current pandemic. This knowledge would be essential not only to inform interventions aimed at preventing and treating psychopathological symptoms among this vulnerable population during and after the pandemic but also to allow more quick and efficient mental health responses in the future if facing a new public health crisis.

Pregnancy is a period in a woman’s life in which considerable physiological, emotional, and psychological changes occur. Due to the COVID-19 pandemic, pregnant women have been facing particularly stressful and challenging circumstances worldwide, which may contribute to poor mental health outcomes (Motrico et al., 2020; Wardrop & Popadiuk, 2015) as well as to poor pregnancy and infant outcomes. For instance, higher risk of obstetrical complications if developing severe illness after infection, impact on pre-existing conditions such as hypertension, and increased risk of preterm birth or fetal growth restriction (Poon et al., 2020).

Portugal was among the European countries with a higher number of COVID-19 infection cases (Tallon et al., 2020). Several changes and restrictions were established in the country in regard to prenatal care (e.g., different timelines for prenatal care appointments and ultrasounds; lack of presence and support from partners during prenatal consultations, labor, and delivery; quarantine time between the infected mother and the newborn). As in other countries, increasing concerns related to COVID-19 (e.g., fear of contracting the virus and/or passing it to the fetus/baby, uncertainty about prenatal care appointments, changes in birth plan expectations, lack of knowledge regarding the impact and consequences of COVID-19 during pregnancy, ambivalence regarding vaccination) (Almeida et al., 2020) and the restrictive measures imposed (e.g., social isolation, individual protective measures) could contribute to higher levels of sadness, loneliness, grief, and/or disappointment (Farewell et al., 2020; Molgora & Accordini, 2020).

According to previous research, contingencies such as those imposed by the pandemic on the perinatal population are likely to produce additional stress and increased risks to pregnant women’s mental health (Berthelot et al., 2020) that may endure for some time and be intensified by new challenges in postpandemic times. Studies performed in diverse countries (Lebel et al., 2020; López-Morales et al., 2021) have shown a high incidence of distress (Berthelot et al., 2020), anxiety (Kotabagi et al., 2020; Liu et al., 2021), and depressive symptoms (Dong et al., 2021; Sun et al., 2020) among pregnant women during the COVID-19 pandemic. Additionally, studies comparing mental health outcomes among the perinatal population before and during the COVID-19 outbreak suggested that the pandemic intensified anxiety and depressive symptoms of pregnant and postpartum women (Ayaz et al., 2020; Zanardo et al., 2020) and, consequently, contributed to the development of mental health problems (Brooks et al., 2020; Davenport et al., 2020).

Among the different mental health indicators assessing the impact of the pandemic in women during the perinatal period, the presence of depressive symptoms has deserved special attention in the literature. Two systematic reviews have shown a high incidence of depressive symptoms among perinatal women, with a prevalence ranging from 30 to 58% in studies conducted in several countries (Ahmad & Vismara, 2021; Yan et al., 2020). Studies have suggested that this prevalence is higher during the pandemic than before the pandemic (Ahmad & Vismara, 2021; Zheng et al., 2021). In Portugal, a recent study found that 26.3% of a sample of pregnant women presented clinically significant depressive symptoms (Padez Vieira et al., 2022). These results are of particular concern considering that an association has been found between depressive symptoms during the perinatal period and impaired mother–infant bonding (Fernandes et al., 2021).

Furthermore, when depressive symptoms are experienced during pregnancy, they may result in a higher risk of low birth weight, preterm birth, intrauterine growth restriction, and pregnancy complications (Grigoriadis et al., 2013), and they may increase the risk and severity of depressive symptoms during the postpartum period (Milgrom et al., 2008; Robertson et al., 2004). In the context of the pandemic, this last aspect may be particularly critical for parents’ adaptation to parenthood and children’s development (Jackson et al., 2021; Venta et al., 2021). The greater lack of social support and difficulties and fears in accessing health care services during the postpartum period—namely, those related to mental health, as well as the greater exclusivity that parental figures assume in infant care due to social isolation during the pandemic—are among some of the reasons for increasing intervention efforts to prevent and treat depressive symptoms during pregnancy when facing public health crises such as the COVID-19 pandemic.

However, the psychological mechanisms that may explain depressive symptoms among this specific vulnerable population during the COVID-19 pandemic remain unclear. Most of the studies focused on the psychological impact of the COVID-19 pandemic in terms of the incidence of psychological symptoms, risk factors (e.g., Liu et al., 2021), or protective factors for maternal mental health, such as social support and physical exercise (Ahmad & Vismara, 2021; Harrison et al., 2021). In fact, few studies performed during the COVID-19 pandemic focused on the psychological processes explaining pregnant women’s depressive symptoms, and to our knowledge, none considered the role of dysfunctional attitudes toward motherhood.

Dysfunctional attitudes toward motherhood are conceptualized as mothers’ beliefs about motherhood and about the maternal role that encompass themes of failure, personal inadequacy, and a sense of hopelessness about the self, the world, and the future (Costa et al., 2018; Sockol et al., 2014). According to the cognitive model of depression, dysfunctional beliefs are considered a cognitive factor that confers vulnerability to the development and maintenance of depressive symptoms (Beck, 1987; Beck et al., 1979), in this case, during the perinatal period (Sockol et al., 2014; Sockol & Battle, 2015). Specifically, it seems that dysfunctional attitudes and beliefs surrounding unrealistic expectations of motherhood and perceived criticism from others about mothering play an important role in the development of depressive symptoms and psychological distress (Fonseca & Canavarro, 2018) and may contribute to self-criticism (Besser & Priel, 2003) and lack of self-care (Vik & Hafting, 2012).

According to previous studies, negative attitudes about motherhood during pregnancy can be particularly activated in the presence of intense stressors (Beck & Haigh, 2014; Sockol et al., 2014), such as public health crises like the COVID-19 pandemic. These negative attitudes may diminish pregnant women’s well-being and, consequently, impact their mental health and the way they will act as mothers. A study by Ravaldi et al. (2021) on the psychological effects of the pandemic on pregnant women found a significant change in women’s expectations of pregnancy and childbirth and an increase in their concerns and distress, particularly if they had a previous history of psychological distress. The authors suggested that the pandemic increased women’s feelings of loneliness and isolation, and this, consequently, activated or worsened their dysfunctional attitudes and beliefs toward motherhood and led to self-criticism (Ravaldi et al., 2021).

Although there is some evidence that supports an association of dysfunctional attitudes and beliefs toward motherhood and depressive symptoms during pregnancy, a better understanding of the nature of this association could enhance early interventions and contribute to the development of preventive interventions to help pregnant women manage their pre-existing dysfunctional beliefs in crisis situations such as the COVID-19 pandemic. According to several studies, an effective way to prevent and treat depressive symptoms during pregnancy is the promotion of mental resources such as self-compassion (Fourianalistyawati et al., 2018; Sacristan-Martin et al., 2019) and mindfulness (Braeken et al., 2017; Goodman et al., 2014). Self-compassion has been described as one’s ability to have a kind, tolerant, and caring attitude toward one’s own suffering (self-kindness), adopting a nonjudgmental attitude regarding one’s feelings of pain, inadequacy, or failure (mindfulness), while recognizing that these experiences are part of a common human condition (common humanity) (Neff, 2003, 2009, 2012). The mindfulness component of self-compassion refers to taking a balanced view of one’s failures, personal suffering, and self-relevant experiences rather than exaggerating or suppressing them (Neff & Germer, 2013). In accordance with previous studies, lower levels of self-compassion may be associated with higher levels of negative self-narratives, depressive symptoms, and less psychological well-being among pregnant women (Felder et al., 2016; Fourianalistyawati et al., 2018).

Moreover, research has suggested that higher self-compassion during pregnancy can have a protective effect against the development of postpartum depression (Cohen, 2010), particularly in the presence of dysfunctional attitudes toward motherhood (Fonseca & Canavarro, 2018). In fact, postpartum women with higher levels of self-compassion seem to face their dysfunctional beliefs about motherhood in a less judgmental and critical way (Fonseca & Canavarro, 2018). A study by Sirois et al. (2019) in a sample of parents with children up to 12 years old reported that higher levels of self-compassion could reduce feelings of guilt and shame and consequently decrease levels of self-criticism, improving their well-being when dealing with the challenges of parenting.

A recent study showed that self-compassion is also a predictor of self-care (Miller et al., 2019). According to Neff (2003, 2009, 2012), self-compassion helps to generate positive attitudes of kindness, understanding, and care that help people to cope with negative emotions, behaviors, and situations and allow a sense of caring. Therefore, it is plausible to expect that pregnant women who have higher levels of self-compassion may also have a greater engagement in self-care practices, such as those associated with mindfulness practices.

According to previous studies, mindfulness practices, such as mindful self-care, can also contribute to increased emotional well-being during pregnancy. Mindful self-care is conceptualized as a process that comprises a mindful awareness and assessment of intrinsic needs and external demands and an intentional commitment to practices of self-care (e.g., healthy and intuitive eating, exercise, yoga, rest, intentionally spending time with friends) to improve well-being and personal efficiency (Cook-Cottone & Guyker, 2018; Hotchkiss & Cook-Cottone, 2019). According to Webb et al. (2019), mindful self-care is negatively associated with depressive symptoms among women in the perinatal period.

Despite some evidence on the effects of self-compassion and mindful self-care on depressive symptoms in the perinatal period, the literature is scarce and focused mainly on other populations (e.g., Cook-Cottone, 2016; Hotchkiss & Cook-Cottone, 2019). Further studies are also needed to improve the understanding of the relationship between maternal attitudes toward motherhood and depressive symptoms, especially in pregnant women during the COVID-19 pandemic.

This study focused on examining the explicative mechanisms of the association between dysfunctional beliefs and attitudes about motherhood and depressive symptoms during pregnancy. Specifically, the present study aimed to analyze whether self-compassion and mindful self-care could play a sequential mediating role in the relationship between dysfunctional attitudes toward motherhood and depressive symptoms in Portuguese pregnant women during the COVID-19 pandemic. Based on previous research, we hypothesized that higher levels of dysfunctional beliefs and attitudes about motherhood would be associated with higher levels of depressive symptoms through lower levels of self-compassion and consequent lower levels of mindful self-care.

## Method

### Participants

A total of 244 pregnant women with a mean age of 32.52 years (*SD* = 4.47) participated in this study. Table 1 describes the sociodemographic, obstetric, and clinical characteristics of the participants in detail. As presented in Table 1, most participants were married or in a relationship, had university or postgraduate studies, and were on paid leave due to pregnancy-related risks. The mean gestational age was 24.97 weeks (*SD* = 8.11), and approximately 67% of the women were going to have their first child.

**Table 1:** Participants’ characteristics

	Total sample (*n* = 244)
Sociodemographic characteristics	
*Age (years), M (SD)*	32.52 (4.47)
*Educational level, n (%)*	
Basic education	4 (1.6)
Secondary education	39 (16.0)
University degree	100 (41.0)
Postgraduate degree	101 (41.4)
*Professional status, n (%)*	
Working	95 (38.9)
Paid leave due to pregnancy-related risk	111 (45.5)
Unemployed	16 (6.6)
Student	6 (2.5)
Other	16 (6.5)
*Marital status, n (%)*	
Married or in a relationship	225 (92.2)
Single or divorced	19 (7.8)
*Relationship length (years), M (SD)*	8.63 (5.09)
*Number of children, n (%)*	
I am having my first child	164 (67.2)
One	61 (25.0)
More than one	19 (7.8)
Household monthly income, n (%)*	
Up to 500€	4 (1.6)
500–1000€	40 (16.4)
1000–2000€	105 (43.0)
2000–3500€	75 (30.7)
More than 3500€	20 (8.2)
*Residence, n (%)*	
Urban	172 (70.5)
Rural	72 (29.5)
Obstetric characteristics	
*Gestational age (weeks), M (SD)*	24.97 (8.11)
*Medical complications during pregnancy, n (%)*	
Yes	42 (17.2)
No	202 (82.8)
*Previous pregnancies, n (%)*	
Yes	107 (43.9)
No	133 (56.1)
Clinical characteristics	
*Current physical health problem, n (%)*	
Yes	43 (17.6)
No	201 (82.4)
*History of psychiatric or psychological problems, n (%)*	
Yes	95 (38.9)
No	149 (61.1)
*COVID-19-related questions*	
*Current or previous infection by COVID-19, n (%)*	
Yes	11 (4.5)
No	233 (95.5)
*Current or previous infection by COVID-19 of someone from social network, n (%)*	
Yes	48 (19.7)
No	196 (80.3)
*Death of someone close by COVID-19, n (%)*	
Yes	6 (2.5)
No	238 (97.5)
*Change in routine prenatal care due to COVID-19, n (%)*	
Yes	126 (51.6)
No	118 (48.4)

The Portuguese minimum wage in 2021 was 665 EUR (787 USD)

### Procedure

This study is part of a wider research project whose main goal was to develop and evaluate the preliminary effectiveness of a brief psychological intervention (“Mind the Mom”), delivered through a mobile application, for pregnant women during the COVID-19 pandemic in Portugal. This research project comprised two assessment moments: (1) a baseline assessment, in which outcome variables were assessed, and (2) a post-intervention assessment, in which outcome variables were reassessed and the Mind the Mom program’s feasibility and acceptability were evaluated. For this study, only the baseline assessment data were used. This study is not preregistered.

Data from pregnant women were collected through a cross-sectional online survey between October 2020 and April 2021. The inclusion criteria to participate were being pregnant, being 18 years or older, having smartphone and Internet access, and having the ability to read and speak Portuguese. The participants’ recruitment occurred through online advertisements on social media websites (e.g., Facebook and Instagram) and on websites focusing on maternity themes. Information about the study’s goals, the voluntary nature of the participation, and the possibility to drop out of the study at any time, as well as the guarantee of anonymity and confidentiality, were explained to the participants in a web form hosted in LimeSurvey®. Only those who gave their informed consent (by answering affirmatively to the question “Do you agree to participate in this study?”) and agreed to the study’s conditions completed the baseline assessment.

### Measures

Participants’ sociodemographic (e.g., age, number of children, marital status, educational level, professional status, average monthly income, and residence), obstetric (e.g., gestational age, previous pregnancies, pregnancy complications), and clinical (e.g., psychopathology history, current or past psychological/psychiatric treatment) information was collected through a self-report form.

The *Edinburgh Postnatal Depression Scale* (EPDS; Cox et al., 1987; Areias et al., 1996) was used to assess the presence of depressive symptoms. The EPDS is a 10-item scale using a 4-point Likert scale assessing women’s experience of the last 7 days concerning several depressive symptoms (e.g., sadness and tearfulness). The total score varies between 0 and 30, with a score higher than 9 indicating the presence of clinically relevant depressive symptoms (Areias et al., 1996). The Portuguese validation studies found good internal consistency (𝛼 = 0.85) and adequate validity. In our sample, Cronbach’s alpha was 0.87 and McDonald’s omega was 0.88.

The *Attitudes Toward Motherhood* (AToM; Sockol et al., 2014; Costa et al., 2018) scale was used to evaluate dysfunctional motherhood-related beliefs. AToM comprises 12 items answered on a 6-point Likert scale (from 0 = *always disagree* to 5 = *always agree*), and it is organized in three dimensions: beliefs related to others’ judgments (e.g., “If my baby is crying, people will think I cannot care for him/her properly”), beliefs related to maternal responsibility (e.g., “Good mothers always put their baby’s needs first”), and beliefs related to maternal role idealization (e.g., “It is wrong to have mixed feelings about my baby”). Higher scores suggest more dysfunctional attitudes toward motherhood. The Portuguese version presents good levels of internal consistency (𝛼 = 0.84 for the total scale) and was found to be a reliable instrument (Costa et al., 2018). In our sample, Cronbach’s alpha was 0.87 and McDonald’s omega was 0.87.

The *Mindful Self-Care Scale—Brief* (MSCS-B; Hotchkiss & Cook-Cottone, 2019) was used to assess six domains of self-care: mindful relaxation, physical care, self-compassion and purpose, supportive relationships, supportive structure, and mindful awareness. The MSCS-B is comprised of 24 items (e.g., “I kindly acknowledged my own challenges and difficulties”) answered on a 5-point Likert scale (ranging from 1 = *never* to 5 = *regularly*). Although the MSCS-B has a factor structure with six factors, it is also possible to compute a total score, which was used in the present study. The higher the total score was, the higher the level of mindful self-care. In our sample, Cronbach’s alpha was 0.91 and McDonald’s omega was 0.90.

The *Self-Compassion Scale—Short Form* (SCS-SF; Raes et al., 2011; Castilho et al., 2015) was used to assess women’s self-compassion levels. The SCS-SF is a 12-item instrument (e.g., “I try to see my failings as part of the human condition”) with a 5-point response scale (from 1 = *almost never* to 5 = *almost always*). Higher scores indicate higher levels of self-compassion. The Portuguese version of the scale is a reliable measure of self-compassion and presents high levels of internal consistency for the total score (α = 0.86). In our sample, Cronbach’s alpha was 0.91 and McDonald’s omega was 0.91.

### Data Analyses

Data analyses were conducted using the Statistical Package for the Social Sciences (IBMS SPSS, version 25.0). Descriptive statistics were calculated to characterize the sample and to describe the study variables (i.e., dysfunctional beliefs toward motherhood, self-compassion, mindful self-care, and depressive symptoms). Pearson’s correlations were computed to examine the associations between sample characteristics and depressive symptoms and between the study variables.

A serial mediation model was tested using the PROCESS macro from SPSS (Hayes, 2018) to examine the direct and indirect effects of dysfunctional attitudes toward motherhood on depressive symptoms through self-compassion and mindful self-care. Model 6 was applied, with dysfunctional beliefs being the predictor variable and depressive symptoms being the outcome variable; self-compassion and mindful self-care were introduced as mediators, in this order. To test for the significance of indirect effects, a bootstrapping procedure was used (5000 bootstrap samples), and 95% bias-corrected confidence intervals (CIs) were calculated. Indirect effects were considered significant if the zero value did not fall within the lower and upper CIs.

## Results

Depressive symptoms were negatively associated with household income (*r* = −0.17, *p* = 0.008) and positively associated with a previous history of psychiatric or psychological problems (*r* = 0.20, *p* = 0.002). These variables were included as covariables in the sequential mediation model. Age, marital status, relationship length, number of children, educational level, professional status, type of residence, current physical health problems, and COVID-related variables were not significantly associated with depressive symptoms.

As shown in Table 2, dysfunctional attitudes toward motherhood were negatively associated with self-compassion and mindful self-care and positively associated with depressive symptoms. Self-compassion was positively associated with mindful self-care and negatively associated with depressive symptoms. Mindful self-care was negatively associated with depressive symptoms.

**Table 2:** Descriptive statistics and Pearson’s correlations between the study variables

**Scales**	*M (SD)*	Correlations between scales		
		SCS-SF	MSCS-B	EPDS
AToM	1.89 (0.93)	−0.43*	−0.36*	0.36*
SCS-SF	2.99 (0.71)	-	0.56*	−0.54*
MSCS-B	18.81 (3.66)	-	-	−0.54*
EPDS	9.80 (4.85)	-	-	-

*p < 0.001

*EPDS*, Edinburgh Postnatal Depression Scale; *AToM*, Attitudes Toward Motherhood scale; *MSCS-B*, Mindful Self-Care Scale—Brief; *SCS-SF*, Self-Compassion Scale—Short Form

**Fig. 1:**
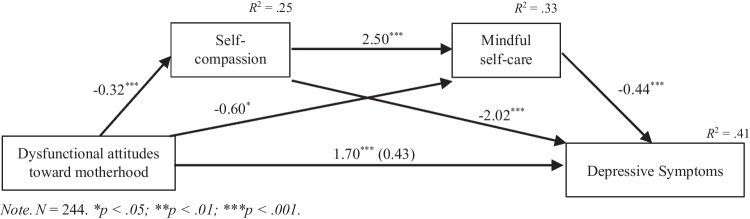
Mediation model. Statistical diagram of the serial mediation model for the presumed association between dysfunctional attitudes toward motherhood and depressive symptoms through self-compassion and mindful self-care while controlling for household income and previous history of psychological or psychiatric problems. Path values represent unstandardized regression coefficients. In the arrow linking dysfunctional attitudes toward motherhood and depressive symptoms, the value without parentheses represents the total effect of the attitudes toward motherhood on depressive symptoms before the inclusion of the mediators. The value inside parentheses represents the direct effect of attitudes toward motherhood on depressive symptoms after controlling for the mediators. Mediators: self-compassion and mindful self-care

Three significant indirect effects were found in the association between dysfunctional attitudes toward motherhood and depressive symptoms: **a** through self-compassion (*point estimate* = 0.65, *SE* = 0.17, *CI* = 0.35/1.02), **b** through mindful self-care (*point estimate* = 0.26, *SE* = 0.14, *CI* = 0.01/0.53), and **c** through self-compassion and mindful self-care (*point estimate* = 0.36, *SE* = 0.09, *CI* = 0.21/0.55). There was no significant direct effect of dysfunctional attitudes toward motherhood on depressive symptoms after controlling for the mediators (*point estimate* = 0.42, *SE* = 0.30, *CI* = −0.14/1.01). Household income (*b* = −0.75, *p* = 0.007) was also found to be associated with depressive symptoms. The serial mediation model was significant (*F*_5,238_ = 32.78, *p* < 0.001) and explained 41% of the variance in depressive symptoms. All paths for the full process model as well as unstandardized regression coefficients are illustrated in Fig. 1.

## Discussion

In the present study, we aimed to investigate whether dysfunctional attitudes toward motherhood were associated with depressive symptoms in pregnant women during the COVID-19 pandemic and whether this relationship was sequentially mediated by self-compassion and mindful self-care. The results showed that the effect of dysfunctional attitudes toward motherhood on depressive symptoms in pregnant women was mediated by self-compassion and mindful self-care, thus corroborating our hypothesis. Our results were congruent with prior studies (Sockol et al., 2014; Sockol & Battle, 2015) showing that more dysfunctional attitudes toward motherhood were associated with more depressive symptoms during pregnancy. Women with dysfunctional attitudes toward motherhood (typically characterized by themes of failure, personal inadequacy, and a sense of hopelessness about the self, the world, and the future) seem to have a more negatively biased interpretation of daily life situations and parenting-related events. Consequently, this seems to lead them to have more negative automatic thoughts and cognitive biases that may increase their depressive symptoms (Fonseca & Canavarro, 2018; Sockol et al., 2014). It seems reasonable to assume that the uncertainty and fears caused by the lockdown situation and the COVID-19 pandemic–related restrictions (e.g., social isolation, lack of social support, postponement of medical appointments) may have emotionally and psychologically overwhelmed pregnant women in their expectations about adjusting to motherhood, which, in turn, seems to be associated with depressive symptoms experienced during pregnancy.

Our findings were also congruent with the previous literature (Costa et al., 2018; Fonseca & Canavarro, 2018) regarding the negative association between dysfunctional attitudes toward motherhood and self-compassion. According to our findings, more dysfunctional attitudes toward motherhood may be associated with lower levels of self-compassion. This may be because pregnant women with dysfunctional attitudes toward motherhood who had to deal with all the changes related to the pregnancy itself and with all the additional challenges imposed by the COVID-19 pandemic may doubt their personal value as mothers, increasing their tendency to judge themselves harshly and consequently increasing their self-criticism. Nevertheless, it is also important to consider that lower levels of self-compassion in pregnancy can lead to a perception of lower maternal self-efficacy and, consequently, lead pregnant women to have more dysfunctional attitudes about motherhood. Or, it might also be considered that other factors (e.g., childhood maltreatment) could affect women’s dysfunctional attitudes about motherhood and women’s self-compassion itself. In line with this, a negative association between dysfunctional attitudes toward motherhood and mindful self-care was also found.

According to our findings, dysfunctional attitudes or beliefs toward motherhood could contribute to reduce women’s commitment to and engagement in self-care practices such as mindful self-care. Further research is needed to better understand this association; it seems that women with more dysfunctional attitudes toward motherhood were less able to use mindful self-care tools to protect themselves from stress and demands of the pandemic, probably hindering their well-being. Considering the Gilbert model (Gilbert, 2005, 2009, 2010), the activation of the threat system occurs when individuals face potentially threatening situations. The COVID-19 pandemic could contribute to the underactivation of the soothing–affiliative system and overstimulation of the threat system of pregnant women with dysfunctional attitudes toward motherhood, leading them to be more self-critical and unable to act in accordance with their self-imposed standards about motherhood, contributing to lower levels of self-compassion, difficulty in engaging in mindful self-care practices, and, consequently, increased depressive symptoms.

In our study, self-compassion was positively associated with mindful self-care. Pregnant women who have higher levels of self-compassion and therefore have a nurturing and caring relationship toward themselves seem to also be more prone to engage in mindful self-care practices (e.g., healthy and intuitive eating, exercise, yoga, rest, and intentionally spending time with friends), probably because they are more able to generate positive attitudes of kindness, understanding, and care that help them cope with negative emotions, behaviors, and situations. Additionally, women with lower levels of self-compassion may be more critical toward themselves and feel guilty when involved in self-care activities; such activities are usually viewed by some mothers with dysfunctional beliefs toward motherhood as selfish, and they may feel unworthy of such care.

A negative association between self-compassion and depressive symptoms was also found. Literature has shown that self-compassion is an indicator of psychological health, can improve quality of life, and has an important role in reducing levels of depression. This is consistent with previous studies (Fourianalistyawati et al., 2018; Mohamadirizi & Kordi, 2016) and suggests that self-compassion may protect pregnant women against depressive symptoms in response to significant stress challenges, such as the COVID-19 pandemic. In this context, if pregnant women with dysfunctional attitudes toward motherhood can experience higher levels of self-compassion, they could be less critical and judgmental about themselves and better manage their dysfunctional attitudes, which could subsequently protect them from the development of and/or an increase in depressive symptoms. Finally, in line with the results found in a study by Webb et al. (2019), our results showed a negative association between mindful self-care and depressive symptoms. It seems that a regular and intentional practice of mindful self-care could improve pregnant women’s physical and psychological well-being (Cook-Cottone & Guyker, 2018; Hotchkiss & Cook-Cottone, 2019). On the other hand, it might be considered that pregnant woman’s physical and psychological well-being could also improve their interest in self-care. In the context of COVID-19, the practice of mindful self-care could help pregnant women maintain a balance between self-needs and external demands, thus reducing depressive symptoms and improving well-being.

Moreover, our study added to previous research showing that self-compassion and mindful self-care fully explained the association between pregnant women’s dysfunctional attitudes toward motherhood and depressive symptoms during the COVID-19 pandemic. According to these findings, it seems reasonable to expect that if pregnant women with dysfunctional attitudes and beliefs toward motherhood can have more compassionate attitudes toward themselves, then they would also be more able to engage in additional practices of mindful self-care and could experience fewer depressive symptoms. However, it is also important to note that self-compassion and mindful self-care practices mediated the association between dysfunctional attitudes toward motherhood and depressive symptoms both sequentially and individually.

We can suggest that pregnant women with more dysfunctional attitudes and beliefs toward motherhood tend to have lower levels of self-compassion. Consequently, they may experience low self-worth, a low sense of self-efficacy, and a lack of self-confidence and think less of themselves (Neff & Vonk, 2009), thus experiencing higher levels of depressive symptoms. More research is also needed regarding the indirect effect of dysfunctional attitudes toward motherhood on depressive symptoms through mindful self-care. Women with dysfunctional attitudes toward motherhood are expected to have difficulties engaging in mindful self-care behaviors that bring awareness and commitment (Cook-Cottone & Guyker, 2018; Hotchkiss & Cook-Cottone, 2019) and that require a shift from a judgmental attitude over concerns with maternity toward loving self-care. These difficulties seem to increase levels of depressive symptoms in these women.

In sum, according to our findings, women with negative thoughts and attitudes toward motherhood who have more self-compassionate attitudes and/or more mindful self-care practices may notice these thoughts and attitudes in a nonjudgmental way and accept them as part of their maternity experience (Pedro et al., 2019). Taking into account the concerns about COVID-19 and the demands of motherhood, if pregnant women with dysfunctional beliefs toward motherhood adopt a nonjudgmental position concerning their attitudes and limitations regarding their role as mothers, accept these limitations as part of the human condition and take part in self-care experiences in times such as the pandemic, they could experience lower depressive symptoms. These findings highlight the importance of considering self-compassion and mindful self-care in psychological interventions to prevent and/or treat depressive symptoms in pregnant women with dysfunctional attitudes and beliefs toward motherhood. This seems to be particularly relevant during stressful public health crises such as the COVID-19 pandemic.

### Limitations and Future Research

This study has some limitations that should be pointed out. The cross-sectional nature of the study does not allow us to draw any causal relationships between these study variables. Future studies using a longitudinal design could overcome this limitation. The sample was self-selected and recruited totally online, so it may not be representative of the Portuguese population. In fact, our sample is composed mostly of women who were married or in a relationship and who were highly educated; furthermore, women without Internet access could not participate. Therefore, future studies should include a more diverse sample, as well as different recruitment methods (e.g., online and face-to-face). Our study included only self-report questionnaires to measure depressive symptoms. Future studies that comprise clinical samples may overcome our limitations to understand whether the mechanisms involved (i.e., self-compassion and mindful self-care) are similar in reducing clinically significant depressive symptoms and whether the associations found in this study are supported. Finally, the validity of the results can be compromised since only self-reported measures were used to assess the study variables. Therefore, participants may be influenced by social desirability and their answers may not reflect their internal experience in a reliable way.

Despite these limitations, this study contributes to the literature in a novel way and provides important research implications. It provides an innovative contribution to better understand specific psychological mechanisms (i.e., self-compassion and mindful self-care) through which mothers’ dysfunctional attitudes and beliefs may exert an effect on depressive symptoms. Specifically, our findings suggest that pregnant women’s dysfunctional beliefs play an important role in the adoption of self-compassion attitudes and/or mindful self-care practices that may prevent mothers from experiencing higher levels of depressive symptoms. Additionally, future research can explore whether there is a mediating effect of these variables (i.e., self-compassion and mindful self-care) on the relationship between dysfunctional attitudes or beliefs toward motherhood and other important and relevant outcomes (e.g., anxiety, stress) in the context of a pandemic, such as COVID-19.

The present study highlights the importance of using specific measures to evaluate cognitive variables among perinatal women because dysfunctional attitudes toward motherhood can constitute a psychological vulnerability for the development of psychological disorders during pregnancy (Beck & Haigh, 2014; Sockol et al., 2014). In line with this, the need to identify pregnant women with dysfunctional attitudes and beliefs toward motherhood is highlighted, as they present a higher risk of developing depressive symptoms. Preventive psychological interventions could aim to challenge pregnant women’s dysfunctional beliefs (e.g., cognitive restructuring) but also include the promotion of a nonjudgmental, mindful (e.g., mindful self-care component), and self-compassionate attitude. Fortunately, self-compassion and mindfulness can be learned through training and meditation, so it can be beneficial to pregnant women to integrate and promote self-compassion and mindful self-care practices in psychological interventions to help them adjust their dysfunctional beliefs and to adopt alternative strategies to manage their thoughts and beliefs. These findings may have relevance for pregnant women who faced various consequences during the COVID-19 pandemic and may be useful in other events/situations that may impose restrictive measures and/or prevent women from accessing health care services.

## Data Availability

The data presented in this study are available on reasonable request from the corresponding author.
